# Haploid yeast cells undergo a reversible phenotypic switch associated with chromosome II copy number

**DOI:** 10.1186/s12863-016-0464-4

**Published:** 2016-12-22

**Authors:** Polina Drozdova, Ludmila Mironova, Galina Zhouravleva

**Affiliations:** 1 Department of Genetics and Biotechnology, St. Petersburg State University, 199034, Universitetskaya nab. 7-9, St. Petersburg, Russia; 2 Laboratory of Amyloid Biology, St. Petersburg State University, 199034, Universitetskaya nab. 7-9, St. Petersburg, Russia

**Keywords:** Aneuploidy, Translation termination, Nonsense suppression, *SUP35*, Yeast, Chromosome instability

## Abstract

**Background:**

*SUP35* and *SUP45* are essential genes encoding polypeptide chain release factors. However, mutants for these genes may be viable but display pleiotropic phenotypes which include, but are not limited to, nonsense suppressor phenotype due to translation termination defect. [*PSI* ^+^] prion formation is another Sup35p-associated mechanism leading to nonsense suppression through decreased availability of functional Sup35p. [*PSI* ^+^] differs from genuine *sup35* mutations by the possibility of its elimination and subsequent re-induction. Some suppressor *sup35* mutants had also been shown to undergo a reversible phenotypic switch in the opposite direction. This reversible switching had been attributed to a prion termed [*ISP* ^+^]. However, even though many phenotypic and molecular level features of [*ISP* ^+^] were revealed, the mechanism behind this phenomenon has not been clearly explained and might be more complex than suggested initially.

**Results:**

Here we took a genomic approach to look into the molecular basis of the difference between the suppressor (Isp^−^) and non-suppressor (Isp^+^) phenotypes. We report that the reason for the difference between the Isp^+^ and the Isp^−^ phenotypes is chromosome II copy number changes and support our finding with showing that these changes are indeed reversible by reproducing the phenotypic switch and tracking karyotypic changes. Finally, we suggest mechanisms that mediate elevation in nonsense suppression efficiency upon amplification of chromosome II and facilitate switching between these states.

**Conclusions:**

(i) In our experimental system, amplification of chromosome II confers nonsense suppressor phenotype and guanidine hydrochloride resistance at the cost of overall decreased viability in rich medium. (ii) *SFP1* might represent a novel regulator of chromosome stability, as *SFP1* overexpression elevates frequency of the additional chromosome loss in our system. (iii) Prolonged treatment with guanidine hydrochloride leads to selection of resistant isolates, some of which are disomic for chromosome II.

**Electronic supplementary material:**

The online version of this article (doi:10.1186/s12863-016-0464-4) contains supplementary material, which is available to authorized users.

## Background

Translation is a very important and energy-demanding process for all living cells including the yeast *Saccharomyces cerevisiae*. Synthesis of ribosomal components is ultimately the main activity of the cell. Apart from copious rRNA molecules produced by RNA polymerases I and III, mRNAs encoding ribosomal proteins and ribosome assembly factors comprise at least 60% of the transcripts produced by RNA polymerase II [1, 2]; in addition, not only ribosome components but many other proteins are required for efficient protein synthesis. No wonder that this system is tightly regulated, and many of its components play a role in this regulation [3].

The process of translation ceases when the translating ribosome encounters one of three stop codons. This step, termed termination, is ensured by the release factors Sup35p (eRF3) and Sup45p (eRF1). These proteins form a complex resembling tRNA; they act by binding to the stop codon and prompting release of the newly synthesized protein to the cytoplasm. Binding of tRNAs and release factor complex to the stop codons exists in a dynamic equilibrium which may shift if levels of any of these components are altered. If the equilibrium shifts in a cell bearing a gene with a premature termination codon (nonsense mutation), the effect of this mutation may be partially compensated for. This phenomenon is referred to as nonsense suppression. The most obvious reasons for nonsense suppression are mutations in tRNA and release factor genes [4]; in addition, partial inactivation of Sup35p deposited in amyloid aggregates ([*PSI* ^*+*^], [5]) was shown to result in a similar phenotype. Mutations in the release factor genes may be adaptive or counter-adaptive depending on the growth conditions, as easily illustrated by a simple example of a *sup35* mutation partially restoring growth of an *ade1-14* mutant strain on adenine dropout media but leading to sensitivity of this strain to elevated temperatures.

Rich collections of spontaneous suppressor mutations in the *SUP35* and *SUP45* genes have been obtained and extensively characterized in several strain backgrounds with different suppressible nonsense mutations [6–9], one of them being 2V-P3982 with *ade1-14* (UGA), *his7-1* (UAA) and *lys2-87* (UGA) and non-suppressor Ade^−^His^−^Lys^−^ phenotype. Two *sup35* strains from this collection were shown to switch from suppressor phenotype (Ade^+^His^+^Lys^+^) to non-suppressor (Ade^±^His^−^Lys^−^) phenotype spontaneously. Curiously, the non-suppressor phenotype was characterized by non-Mendelian inheritance, could be eliminated on media containing guanidine hydrochloride (GuHCl) and re-appeared after GuHCl-caused elimination, similar to known prions [10].

This prion-like determinant associated with reduced nonsense suppression efficiency was designated [*ISP* ^+^] for “**i**nversion of **s**uppressor **p**henotype”. Despite being similar to a prion with a clear link to a transcriptional regulator Sfp1p, a potentially prionogenic protein enriched in asparagine and glutamine residues [11], [*ISP* ^+^] has a number of features which distinguish it from most “canonical” prions. Its propagation does not depend on the Hsp104 chaperone [10], which is required for propagation of other prions [12], and deletion of the *SFP1* gene conveys a phenotype drastically different from the [*ISP* ^+^] phenotype [11]. In addition, [*ISP* ^+^] strains have been found to contain not only the *sup35-25* suppressor mutation, but also a missense substitution *sup45-400*. The combination of *sup35* and *sup45* mutations contributes to the development the suppressor phenotype, since introduction of plasmid-borne wild-type *SUP45* into the [*ISP* ^+^] strain leads to [*isp* ^−^]-like suppressor phenotype [13].

In presence of pre-existing suppressors, translation termination efficiency may be modulated by multiple factors including the *Ψ* factor [14], *i.e.* the [*PSI* ^+^] prion [15], or an additional chromosome [16]. Similar to suppressor mutations and prions, presence of an additional chromosome may confer adaptiveness or counter-adaptiveness depending on the conditions tested [17, 18]. Many natural isolates were shown to be aneuploid [19–21], and in some natural isolates prions were revealed [22, 23], which probably reflects the utility of these traits.

In this work, we show that in an unstable strain copy number of chromosome II can modulate nonsense suppressor phenotype and resistance to guanidinium chloride. Our results also suggest that Sfp1p, a transcriptional regulator, is implicated in maintenance of chromosome stability.

## Results

### Isp^+^ and Isp^−^ isolates used for transcriptional profiling differ in copy number of chromosomes II and IX, and genome sequencing confirms this result

[*ISP* ^+^] has been studied in a group of closely related strains mostly ascending to 25-2V-P3982 (a 2V-P3982 derivative bearing the *sup35-25* mutation [10]). In recent works, the 25-25-2V-P3982 strain was used, which had been derived from 25-2V-P3982 through mating type switch [13]. The strain has been stored in glycerol stocks with several episodes of partial defreezing; so, genetic identity of the original strains of the early 2000s and the strains used in this work cannot be guaranteed. For this reason, throughout this work we will refer to *sup35-25* isolates as either Isp^+^ or Isp^−^. Isp^+^ stands for His^−^Lys^−^ phenotype while Isp^−^ refers to His^+^Lys^+^ phenotype; isolates of both types are Ade^+^.

**Fig. 1:**
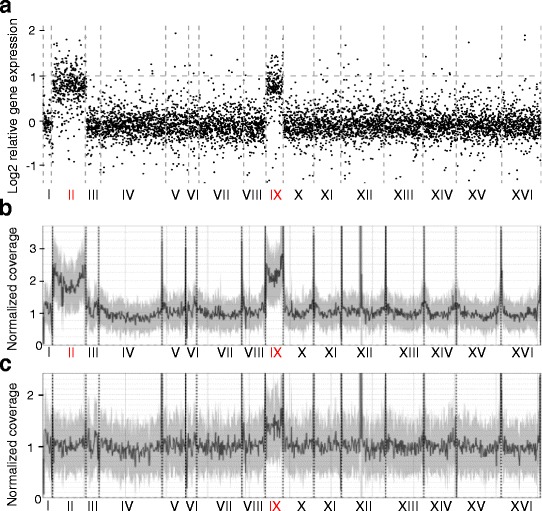
Isp^+^ and Isp^−^ isolates differ in copy number of chromosomes II and IX. **a** Expression values for all the genes in an Isp^−^ isolate (m2) relative to an Isp^+^ one (p2), sorted by chromosome. **b** Normalized coverage throughout the reference genome for an Isp^−^ isolate (m2). **c** Normalized coverage throughout the reference genome for an Isp^+^ isolate (p3). Chromosome numbers are indicated at the bottom. Full datasets are available in Additional file 2: Table S1

Recently, we compared the transcriptional profiles of two isolates presumed to be [*ISP* ^+^] and [*isp* ^−^] [24]. In this work, we will refer to these isolates as p2 (Isp^+^, p for ‘plus’) and m2 (Isp^−^, m for ‘minus’), respectively (see below). We found a small number (*~*300) of differentially expressed genes which fall into two classes, those upregulated in the Isp^+^ isolate and those upregulated in the Isp^−^ one. While genes of the first group formed particular functional clusters associated with nutrient assimilation and metal ion import, the second group of genes was not enriched in any particular functional groups and thus similarly lacked common reason for changing expression [24]. However, genes of the second group were united by another feature, their chromosomal location. Hypergeometric test showed they were significantly enriched in genes located on chromosomes II and IX (*p* = 3 × 10^−118^ and *p* = 2 × 10^−47^, respectively). Plotting relative (Isp^−^/Isp^+^) gene expression values against gene position in the reference genome clearly illustrated the same tendency (Fig. 1a).

In order to check how these data correspond to the previously reported gene expression data for disomic strains, we exploited the vast body of evidence generated for twelve different haploid disomic strains and accumulated in the work of Dephoure *et al*. [25]. Indeed, the distribution of gene expression values looked very similar (Additional file 1: Figure S1), even though overall Pearson correlation of the expression profile of the Isp^−^ isolate with expression profiles of strains disomic for only chromosome II or only chromosome IX [25] was not high (*r* = 0.38 and *r* = 0.20, respectively).

**Fig. 2:**
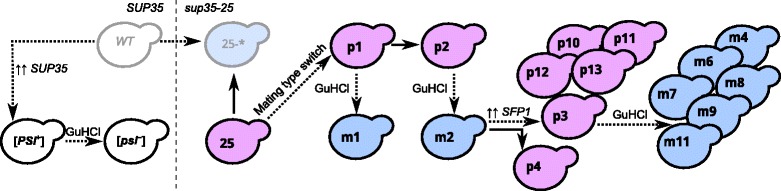
Relationship of the isolates used in this work. Grey contour, clones no longer existing in the collection; black contour, isolates analyzed in our work. The dotted line separates *SUP35 SUP45* and *sup35-25 sup45-400* clones. Blue background signifies Lys^+^ or Lys^+^His^+^ phenotype (Isp^−^, m for ‘minus’); purple background signifies Lys^−^His^−^ (Isp^+^, p for ‘plus’) phenotype. GuHCl, guanidine hydrochloride treatment. Solid arrows signify passaging without treatment; dotted arrows signify GuHCl treatment or transformation (indicated above). ↑↑ *SFP1*, *SFP1* overexpression with subsequent plasmid loss for isolation of Isp^+^ clones. ↑↑ *SUP35*, *SUP35* overexpression with subsequent plasmid loss for [*PSI* ^+^] induction

However, sequencing read depth could provide an additional (and possibly more reliable) measure of chromosome copy number than the mRNA level. It could also indicate whether the isolates (even though unlikely) differ in some point mutations which contribute to the phenotype. Two isolates were chosen for whole genome sequencing. The Isp^−^ isolate was a copy of same Isp^−^ isolate used for transcription profile analysis (m2), which had been passaged on YEPD and then stored at −80 °C as a glycerol stock. The Isp^+^ isolate (p3) was a derivative of m2 obtained with transient *SFP1* overexpression (see Fig. 2). First, depth of coverage analysis was performed (Fig. 1b, c). We indeed found a difference in chromosome II and IX copy number. Surprisingly, the Isp^+^ isolate (p3) showed non-integer coverage for chromosome IX (Fig. 1c), which probably means that it consisted of a mixture of monosomic and disomic cells. In agreement to the gene expression values, the Isp^−^ isolate (m2) showed approximately two-fold higher coverage for chromosomes II and IX than for the other chromosomes (Fig. 1b). In addition, we compared single nucleotide variation data for the two isolates and did not find any “suspicious” positions which could have been selected for; chromosome II of the m2 genome was homozygous for the *sup45-400*, *his7-1* and *lys2-87* alleles.

### Transitions between Isp^+^ and Isp^−^ phenotypes strongly correlate with changes in chromosome II number

As the molecular difference between Isp^+^ and Isp^−^ isolates turned out to be distinct from the prion-like determinant described previously, we asked whether it was also reversible. Thus, it was crucial to establish whether the chromosome copy number difference would be reproduced in newly obtained Isp^+^ and Isp^−^ isolates.

There are two known means to obtain Isp^+^ clones from Isp^−^ ones, namely spontaneous appearance happening at a frequency of about 1 per 10,000 cells [10] and *SFP1* overexpression elevating this frequency ~700-fold [11]. There is only one known way to obtain Isp^−^ isolates from Isp^+^ ones, GuHCl treatment [10]. We employed these three methods and used m2 to obtain one spontaneous Isp^+^ isolate (p4; Fig. 2, lower part) and four more Isp^+^ isolates after transient overexpression of *SFP1* (isolates p10, p11, p12, p13; Fig. 2, lower part); p3 was passaged on GuHCl-containing media five times to select six Isp^−^ isolates (m4, m5, m7, m8, m9 and m11; Fig. 2, lower part). In addition, we used the oldest glycerol stocks available to recover Isp^+^ and Isp^−^ isolates p1 and m1. Finally, we tried to obtain strains parental to 25-25-2V-P3982 (*sup35-25 MAT*a) and used in earlier works, 25-2V-P3982 (*sup35-25 MATα*) and 2V-P3982 (*SUP35 MATα*). 25-2V-P3982 was recovered from glycerol stock (Fig. 2, isolate 25). No stocks of the original 2V-P3982 strain were available but its [*PSI* ^+^] derivative was stored; we recovered it (Fig. 2, isolate [*PSI* ^+^]) and also treated it with GuHCl to obtain a prionless strain (Fig. 2, isolate [*psi* ^−^]).

Then, we employed microarray-based comparative genomic hybridization (aCGH) to infer copy number of each chromosome in each of these isolates (Table 1). Chromosome II copy number perfectly correlates with the Lys^+^/Lys^−^ phenotype of the particular isolate: to sum up, all 7 Isp^+^ isolates tested were monosomic for chromosome II while all 8 Isp^−^ isolates had this chromosome amplified. In addition, we found some variability in copy number of chromosomes I, IX and XIV (Table 1). We could not associate chromosome I or XIV disomy with any changes in nonsense suppression efficiency. Chromosome IX copy number might influence *his7-1* suppression efficiency since m1, the only Isp^−^ isolate monosomic for chromosome IX in our analysis, is characterized by poorer growth on histidine dropout medium than the other Isp^−^ isolates; however, we consider this unlikely as the original Isp^−^ isolate has been previously shown to suppress both *his7-1* and *lys2-87* [10].

**Table 1:** Isp^−^ isolates differ from Isp^+^ ones by chromosome II copy number

Isolate name	Phenotype	Karyotype
[*PSI* ^+^]	Ade^+^His^−^Lys^−^	euploid
[*psi* ^−^]	Ade^−^His^−^Lys^−^	+I
25, p1, p10, p11, p12, p13	Isp^+^	euploid
p3		+XIV
p4		+IX
**m1**	**Isp** ^**−**^	**+II**
**m2, m4, m6, m7, m8, m9, m11**		**+II, IX**

Shown are summarized results of the aGCH analysis. For isolate names, see Fig. 2. Isp^−^ isolates are indicated in bold. For full data, see Additional file 3: Figure S2 and Additional file 4: Table S2

### Possible mechanisms for transitions between the Isp^+^ and Isp^−^ states

**Fig. 3:**
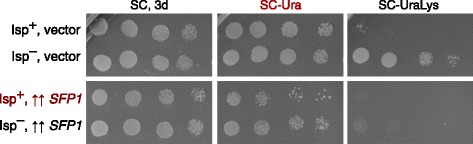
*SFP1* overexpression is slightly more toxic for an Isp^+^ isolate (p1) than for a Isp^−^ one (m1). Shown are five-fold serial dilution starting with equal number of cells after 3 (indicated) or 6 days of incubation. Vector, pRS426. ↑↑ *SFP1*, pRS426-SFP1

Our results show that Isp^+^ clones isolated after transient *SFP1* overexpression lose extra chromosomes. Thus, *SFP1* overexpression might elevate the frequency of extra chromosome loss. The simplest explanation would be that *SFP1* overexpression, which is toxic for the cell, might be even more toxic for disomic cells, allowing for selection of euploids. However, this was not true; moreover, *SFP1* overexpression was even more toxic for Isp^+^ isolates (Fig. 3).

**Fig. 4:**
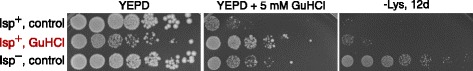
The Lys^+^ phenotype is co-selected with resistance to GuHCl. An Isp^+^ (p1) isolate was passaged on either YEPD (upper line) or YEPD with 5mM GuHCl (middle line) five times; an Isp^−^ isolate (m1) passaged on YEPD (lower line) is shown for comparison. Shown are five-fold serial dilutions starting with equal number of cells after 6 or 12 (indicated) days of incubation

In the case of the reverse transition, Isp^+^ to Isp^−^, selection for resistance to GuHCl may take place. [*isp* ^−^] isolates had been shown to be more resistant to 5mM GuHCl than [*ISP* ^+^] ones [10]; we confirmed this results (compare upper and lower lines at Fig. 4). Curing of prions with GuHCl depends on Hsp104 [26] but the [*ISP* ^+^] prion had been shown to be independent of Hsp104 [10]. What happened when strains were treated with GuHCl is unclear. We passaged an Isp^+^ isolate on either YEPD or YEPD with GuHCl five times and then compared the phenotype (Fig. 4). GuHCl treatment led to selection for GuHCl-resistant isolates in Isp^+^ with simultaneous selection for Lys^+^ (Isp^−^) clones. Thus, amplification of chromosome II might be one of the mechanisms of adjustment to GuHCl.

### Mechanisms for nonsense suppression associated with chromosome II disomy

**Fig. 5:**
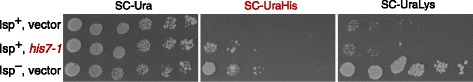
An additional copy of *his7-1* improves growth of an Isp^+^ isolate (p3) on histidine dropout medium. Shown are five-fold serial dilutions for representative clones starting from equal number of cells, after 14 days of incubation. Vector, pRS316. *his7-1*, pRS316-his7-1

As we show, chromosome II ploidy state correlates with the efficiency of suppressor phenotype, *i.e.*, growth on histidine or lysine dropout media. This phenotype might be associated with marker nonsense mutations *his7-1* and *lys2-87* used to assess nonsense suppressor efficiency, as both genes are located on chromosome II. We chose one of these marker genes, *his7-1*, and checked whether introduction of an additional copy of this allele on a centromeric plasmid would produce a result similar to chromosome amplification. Indeed, an additional copy of *his7-1* did elevate growth on histidine dropout medium without affecting growth on lysine dropout medium (20 independent transformants were checked; a representative clone is shown at Fig. 5). However, this effect was not strong enough to mimic the Isp^−^ phenotype (Fig. 5, compare lines 2 and 3). Thus, His^+^ phenotype of the Isp^−^ isolates depends on the *his7-1* copy number, but there might be other contributing factors.

## Discussion

In this work we show that the phenotypic difference between the Isp^+^ and Isp^−^ isolates depends on chromosome II copy number. This finding is unexpected since these isolates were originally described as different in their [*ISP* ^+^] prion state [10, 11]. The original suppressor *sup35-25* isolate was not preserved, so we cannot directly determine whether it was disomic or displayed marked nonsense suppression for another reason. However, progeny of a hybrid of *sup35-25* [*isp* ^−^] and *SUP35* strains had shown monogenic segregation of the suppressor phenotype [6, 10] while a hybrid of *sup35-25* [*ISP* ^+^] and *SUP35* strains showed deviation from 2:2 segregation [10]. If this Isp^−^ strain were disomic for chromosome II and the disomy contributed to the suppressor phenotype, as we see in the strain studied in this work, deviation from monogenic segregation would have been observed in the former, but not in the latter case. These data suggest that the Isp^+^/Isp^−^ phenotypes studied in this work represent phenocopies of the original phenotypes determined by presence or absence of the [*ISP* ^+^] prion.

We have shown that transitions from Isp^+^ to Isp^−^ phenotype and *vice versa* are indeed associated with changes in chromosome II copy number (Fig. 2 and Table 1). However, the question why these transitions happen still remains. Spontaneous appearance of Isp^+^ clones can be attributed to accidental loss of the extra chromosome in cell divisions facilitated by the fact that Isp^+^ clones grow slightly faster than Isp^−^ ones [10]. However, it is unclear why overexpression of *SFP1* elevates the frequency of extra chromosome loss. Increased level of Sfp1p might modulate expression of some target gene regulating chromosome maintenance or Sfp1p itself might interact with such regulator. Chromosome loss can happen due to unrepaired double strand breaks or defects in chromosome segregation. *SFP1* overexpression affects cell cycle, most probably causing cells to pause in G2 [27], so we can speculate that cells overexpressing *SFP1* could either have more double strand breaks or be defective for double strand break repair or spindle assembly.

The reverse transition, from Isp^+^ to Isp^−^, might be associated with selection for GuHCl resistance. It is worth emphasizing that while 5 mM GuHCl inhibits growth of yeast cells (Fig. 4, compare left and middle panels), growth of Isp^+^ isolates is inhibited by 5 mM GuHCl more than the growth of Isp^−^ isolates [10], and we confirm this result (Fig. 4). GuHCl resistance might be caused by increased level of Hal3p (Sis2p) reported earlier [28] or also by some gene located on chromosome II. Emergence of aneuploid clones in response to a stressful condition is similar to other reported cases such as chromosome III amplification in response to heat stress or chromosome V amplification as an adaptation to high pH [17] as well as chromosome XIII disomy making *gal7* strains galactose tolerant [29]. Interestingly, chromosome II disomy has been already described in some laboratory strains as a compensatory mechanism. It was shown to arise in response to polyQ toxicity due to the Sup45 protein [30] or to provide viability for strains deleted for the *hta1-htb1* locus due to the increasing dosage of the *HTA2* and *HTB2* genes [31]. As only some of the clones growing well on GuHCl-containing media are Lys^+^ and further prove to be disomic for chromosome II, we suggest that there are multiple ways to adjust to high GuHCl concentrations toxic for the cell, one of them being chromosome II disomy associated with the Isp^−^ phenotype.

We show that chromosome II copy number (monosomy/disomy) perfectly correlates with the Isp^+^/Isp^−^ phenotype of the particular isolate, suggesting that it is the reason for the difference in growth on histidine or lysine dropout media. The simplest explanation of the mechanism would be increased dosage of the *his7-1* or *lys2-87* mRNA, respectively. We checked this hypothesis and showed that introduction of an additional copy of *his7-1* indeed leads to His^+^ phenotype of an Isp^+^ isolate. We can speculate that the same mechanism takes place in the case of *lys2-87*. However, as an Isp^+^ isolate with two copies of *his7-1* still produces slightly less His^+^ clones than an Isp^−^ isolate (Fig. 5); this might be caused either by the missense mutation in the plasmid-borne *his7-1* copy or by influence of some other gene(s) located on chromosome II or regulated by chromosome II genes. Among such candidate genes could be several tRNA genes or the *sup45-400* allele*,* as introduction of wild-type *SUP45* to Isp^+^ strains has been shown to mimic the Isp^−^ phenotype [13]. To the extent of our knowledge, this is the second reported case of nonsense suppressor phenotype modulated by aneuploidy, the first being chromosome VIII disomy elevating translation termination efficiency due to *SPB1* duplication [16].

Finally, there is a question why many of the isolates checked in this work contain additional chromosomes. Our data do not allow us to estimate the frequency of chromosome gain, but still the proportion of aneuploid isolates in our analysis, the variability of karyotypes and non-integer coverage data suggest significant genome plasticity. The 25-25-2V-P3982 strain possesses missense mutations in *SUP35* and *SUP45*. These genes have been linked to chromosome stability [32], but as we also found additional chromosome I in a *SUP35 SUP45* strain ([*psi* ^−^]), we consider *sup35-25* or *sup45-400* an unlikely cause of the genome instability. In an attempt to find other genes which could be connected to genome instability in 25-25-2V-P3982, we compared the list of 46 genes with premature stop codons found in the genome of this strain [33] to the curated list of 692 chromosome instability genes [32]. Intersection of these lists returned three genes, *ADE1*, *MNL1* and *CNN1*. The *ade1-14* allele has been used as a marker of nonsense suppression in multiple works [26] without reports on genome instability, and in addition, it is partially suppressed in the strain used; thus, we consider *ade1-14* an unlikely contributor to the observed chromosome instability. *MNL1*, encoding a protein residing in the endoplasmic reticulum and acting in glycoprotein degradation [34], was detected to influence genome stability in A-like faker screen [35], but the mechanism behind this effect is unclear. The third gene, *CNN1*, encodes a kinetochore component; deletion of this gene had been associated with chromosome segregation defects in several works [35, 36]. *CNN1* contains a premature stop codon in 25-25-2V-P3982 but not in other related strains of the Peterhof genetic collection [33] and might contribute to the observed chromosome instability.

## Conclusions

1. In our experimental system, amplification of chromosome II confers nonsense suppressor phenotype and guanidine hydrochloride resistance at the cost of overall decreased viability in rich medium.
2. *SFP1* might represent a novel regulator of chromosome stability, as *SFP1* overexpression elevates frequency of the additional chromosome loss in our system.
3. Prolonged treatment with guanidine hydrochloride leads to selection of resistant isolates, some of which are disomic for chromosome II.

## Methods

### Strains and cultivation

P-2V-P3982 (clone [*PSI* ^+^]) (*MAT*α *ade1-14 his7-1 lys2-87 ura3Δ0 thr4-B15 leu2-B2* [*PSI* ^*+*^]) was derived from 2V-P3982 [10] with transient *SUP35* overexpression (Kirill V. Volkov, unpublished data). Clone 25 ascends to the 25-2V-P3982 strain (*MAT*α *ade1-14 his7-1 lys2-87 ura3Δ0 thr4-B15 leu2-B2 sup35-25 sup45-400*) described earlier [10]. 2-P-2V-P3982 (clone [*psi* ^−^]) is derived from P-2V-P3982 with passaging on YEPD medium with 5mM guanidine hydrochloride three times. 25-25-2V-P3982 (*MATa ade1-14 his7-1 lys2-87 ura3Δ0 thr4-B15 leu2-B2 sup35-25 sup45-400*) was described earlier [11, 13, 33]; generation of isolates is summarized in Fig. 2. Either PSL2 [37] or p1 genomic DNA was used as euploid control in aCGH experiments.

Standard yeast media [38, 39] with modifications were used. Guanidine hydrochloride (Sigma G-3272-100G) was added to a final concentration of 5 mM. Yeast strains were cultivated at 26 °C, *E. coli* cells were cultivated at 37 °C.

Yeast transformation was carried out according to the standard protocol [40] with modifications.

### Plasmids

Cloning was carried out according to standard protocols [41, 42]. To obtain pRS316-his7-1, *his7-1* with flanking regions was PCR amplified from genomic DNA of p3 with primers HIS7_F_HindIII_ (GTAACAAGCTTTCTTTCCTCTACCACTGCCAA) and HIS7_R_HindIII_ (ACCATAAGCTTTGGTACAATTTCTCCAAGCTG) and ligated into RS316 [43] at HindIII sites. In addition to a nonsense mutation A299T leading to premature stop codon at position 77 [44], it contains a PCR-induced substitution T947C leading to a missense mutation L316P. pRS426-SFP1, a pRS426 [45] derivative containing *SFP1* under the control of its own promoter, was described earlier [11]. pRS316 [43] and pRS426 [45] were also used as control vectors.

### DNA extraction and analysis

DNA extraction for whole genome sequencing, library preparation and data analysis methods was described earlier [33]. DNA extraction for PCR was performed as described in [38]. DNA extraction for aCGH, labeling and hybridization were performed as described in [46] except that yeast strains were grown at 22 °C for four days. Custom 8x15k design (AMID 028943) Aligent arrays were used; the slides were scanned and then analyzed with GenePix Pro 6.0 software. In each case, phenotype of the clones used for nucleic acid extraction was checked at the moment of extraction with spotting on selective media.

### Data analysis and availability

Microarray data analysis was performed in R [47] with limma [48]; ggplot2 [49] was used for plotting. Bowtie2 [50] was used for short read alignment. Mapping coverage was plotted with Qualimap [51]. aCGH data were analysed with CGH-Miner Excel add-in [52]. Details of next generation sequencing data analysis and expression microarray data analysis are provided in [24, 33], respectively.

## Additional files

Additional file 1: Figure S1. Expression values for all the genes disomic strains relative to wild-type control from [25] compared to expression values of the Isp^−^ isolate (m2) relative to an Isp^+^ one (p2), sorted by chromosome. Analysis as in Fig. 1. (PNG 611 kb)
Additional file 2: Table S1. Expression values for all genes in an Isp^−^ isolate (m2) relative to an Isp^+^ one (p2) and mean coverage in approximately 1kb-long windows for an Isp^−^ isolate (m2) relative to an Isp^+^ one (p3). Genes/windows are sorted by chromosomal position. Genes or windows located on duplicated chromosomes are in bold. (XLS 1215 kb)
Additional file 3: Figure S2. Graphical summary of aCGH results for each isolate tested. (a) Schematic represenation of the results combined with the information on relationship of the strains (see Fig. 2). aCGH, microarray-based comparative genomic hybridization. WGS, whole genome sequencing. (b) Summarized data for each isolate. Horizontal lines represent chromosomes; red signifies amplified regions while green signifies deleted regions. Clones or strains used as experimental and control samples are indicated on the top of each page. (PDF 3621 kb)
Additional file 4: Table S2. Raw aCGH data for each isolate tested. (XLS 3589 kb)

## Abbreviations

aCGH: Microarray-based comparative genomic hybridization
GuHCl: Guanidine hydrochloride
PCR: Polymerase chain reaction
