# Supplementary figures and images for: Haploid yeast cells undergo a reversible phenotypic switch associated with chromosome II copy number
Source: BMC Genet. 2016 Dec 22;17(Suppl 3):152. doi: 10.1186/s12863-016-0464-4 (PMC5249023; doi:10.1186/s12863-016-0464-4)

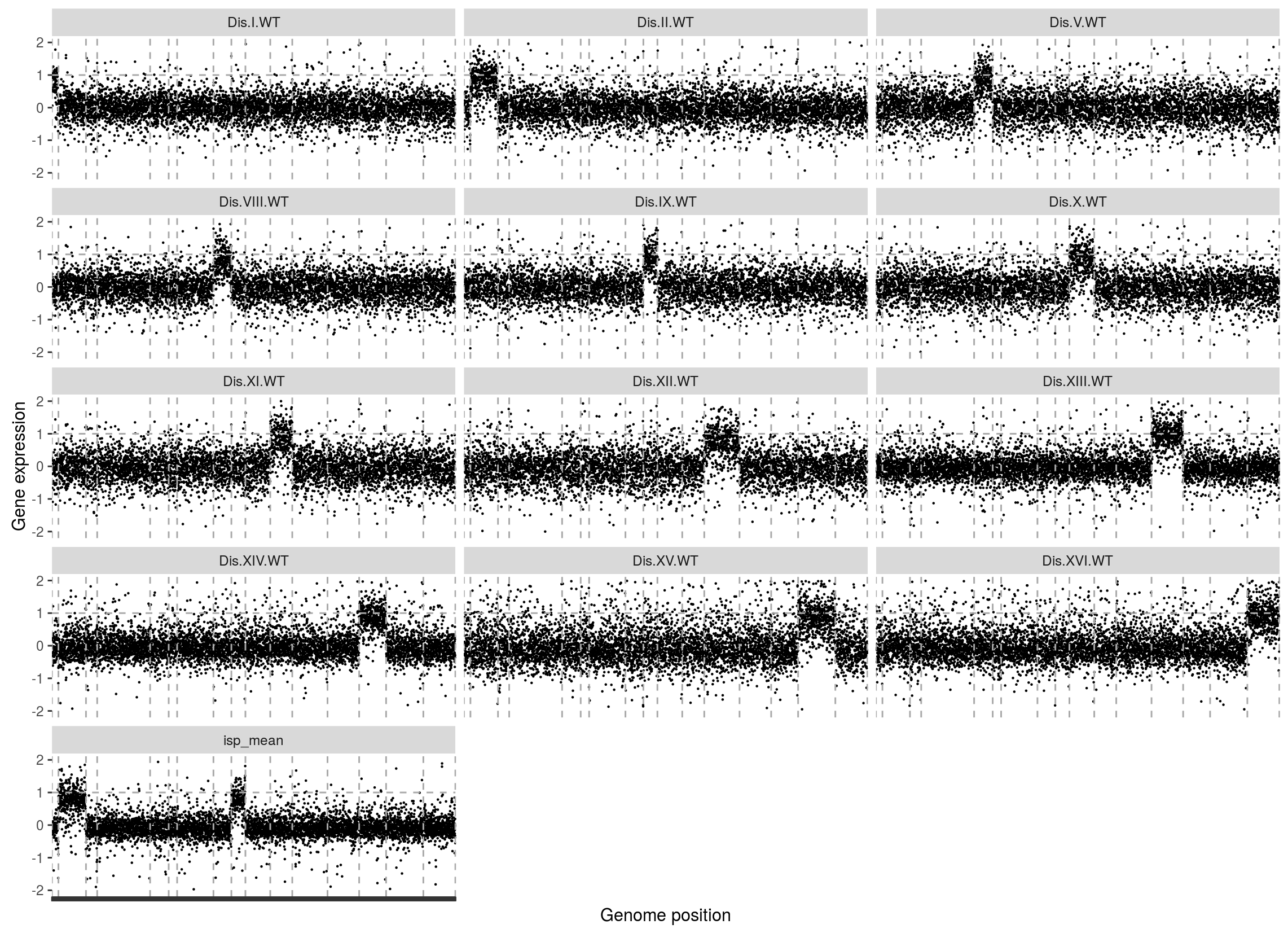

Supplement: Additional file 1: Figure S1. — Expression values for all the genes disomic strains relative to wild-type control from [25] compared to expression values of the Isp− isolate (m2) relative to an Isp+ one (p2), sorted by chromosome. Analysis as in Fig. 1. (PNG 611 kb) [file 12863_2016_464_MOESM1_ESM.png]
