# Supplementary material for: Haploid yeast cells undergo a reversible phenotypic switch associated with chromosome II copy number
Source: BMC Genet. 2016 Dec 22;17(Suppl 3):152. doi: 10.1186/s12863-016-0464-4 (PMC5249023; doi:10.1186/s12863-016-0464-4)

a

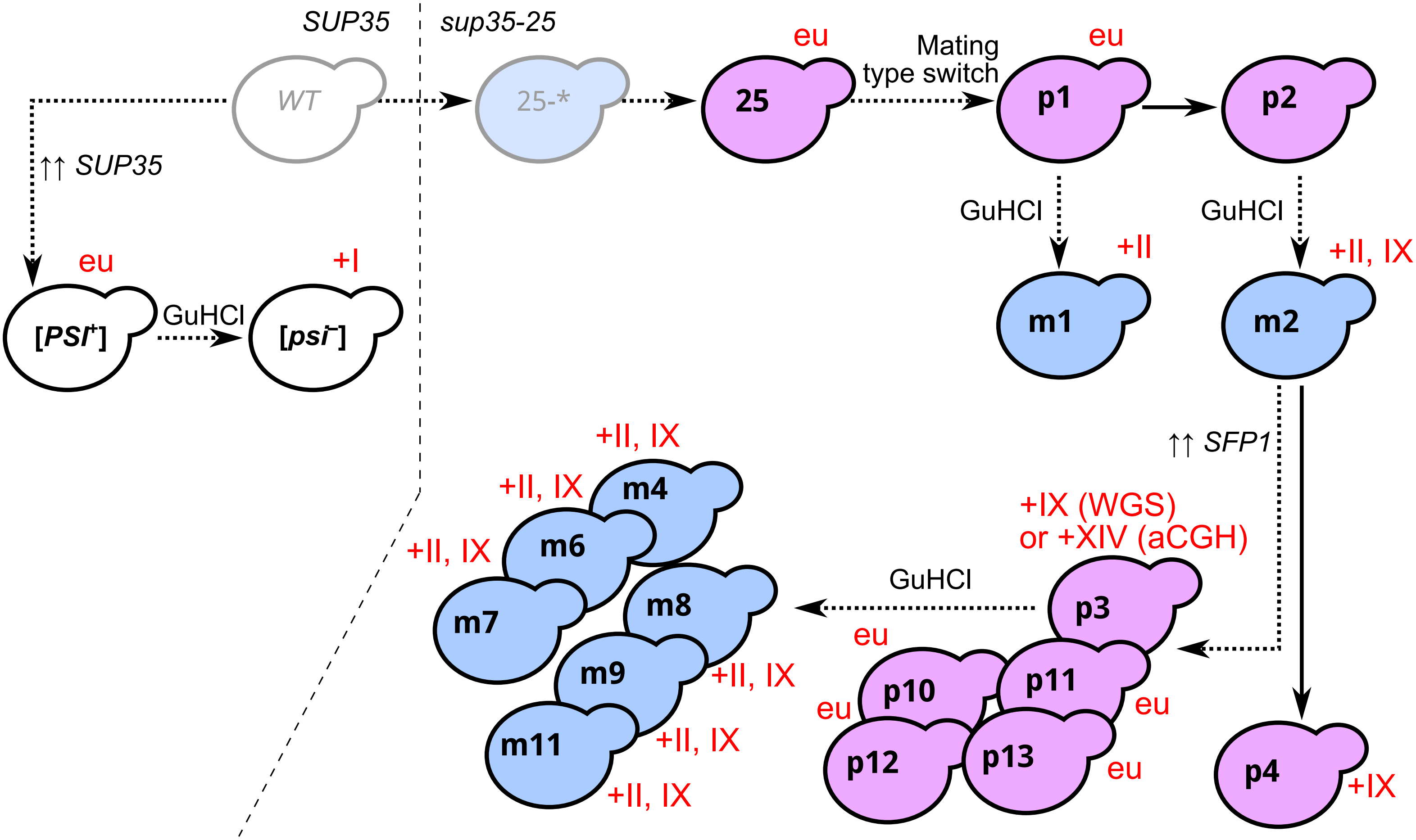

SUP35
sup35-25
WT
25-*
25
eu
Mating type switch
p1
eu
p2
↑↑ SUP35
GuHCl
GuHCl
m1
+II
m2
+II, IX
eu
+I
[PSI+]
GuHCl
[psi−]
↑↑ SFP1
+II, IX
m4
+II, IX
m6
+II, IX
m7
m8
m9
+II, IX
m11
+II, IX
+II, IX
+IX (WGS)
or +XIV (aCGH)
GuHCl
p3
eu
p10
p11
eu
eu
p12
p13
eu
p4
+IX

b

CLAC Plot for Sample: [*PSI*$^+$] vs PSL2

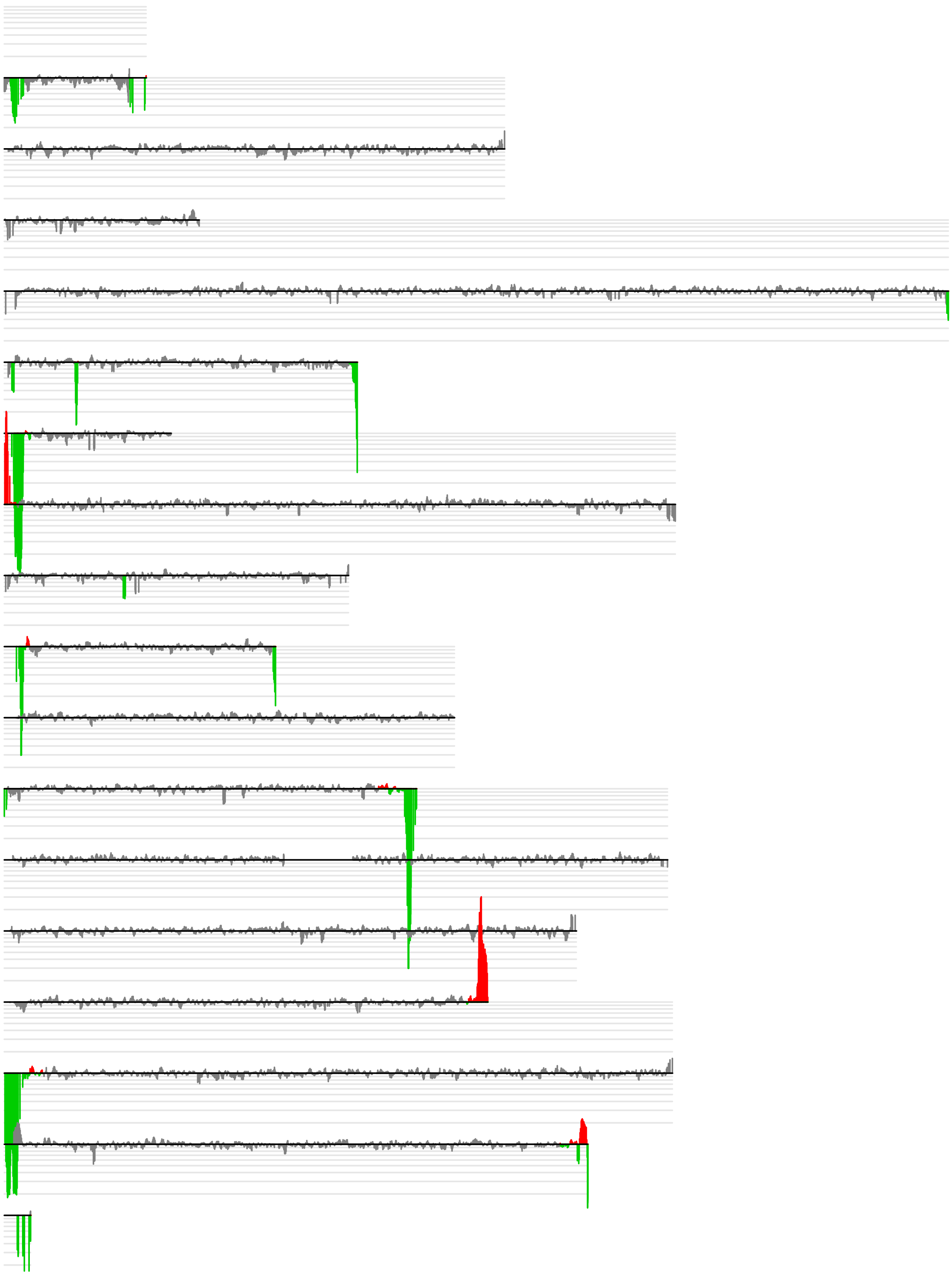

CLAC Plot for Sample: [*psi*⁻] vs PSL2

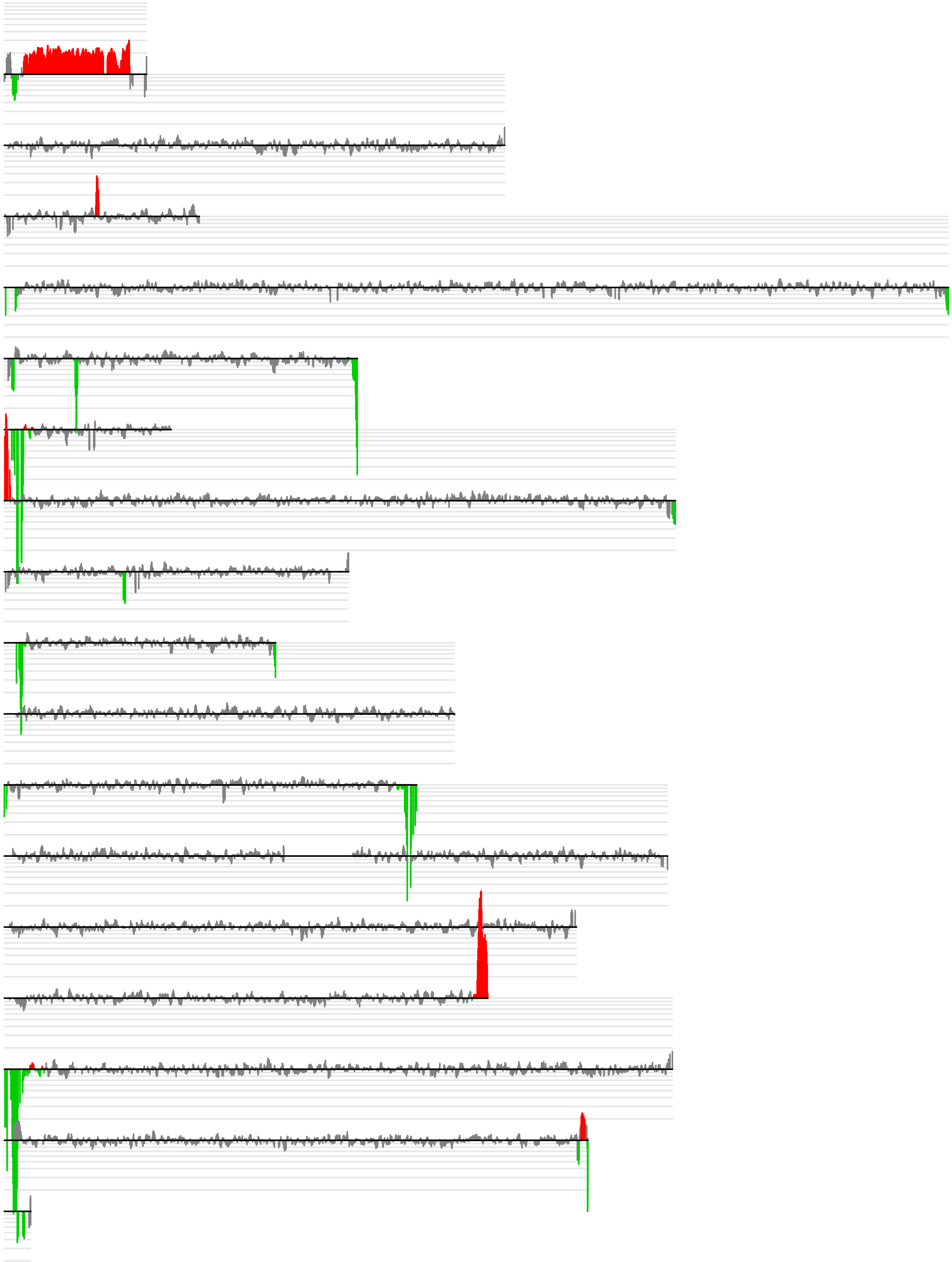

CLAC Plot for Sample: 25 vs PSL2

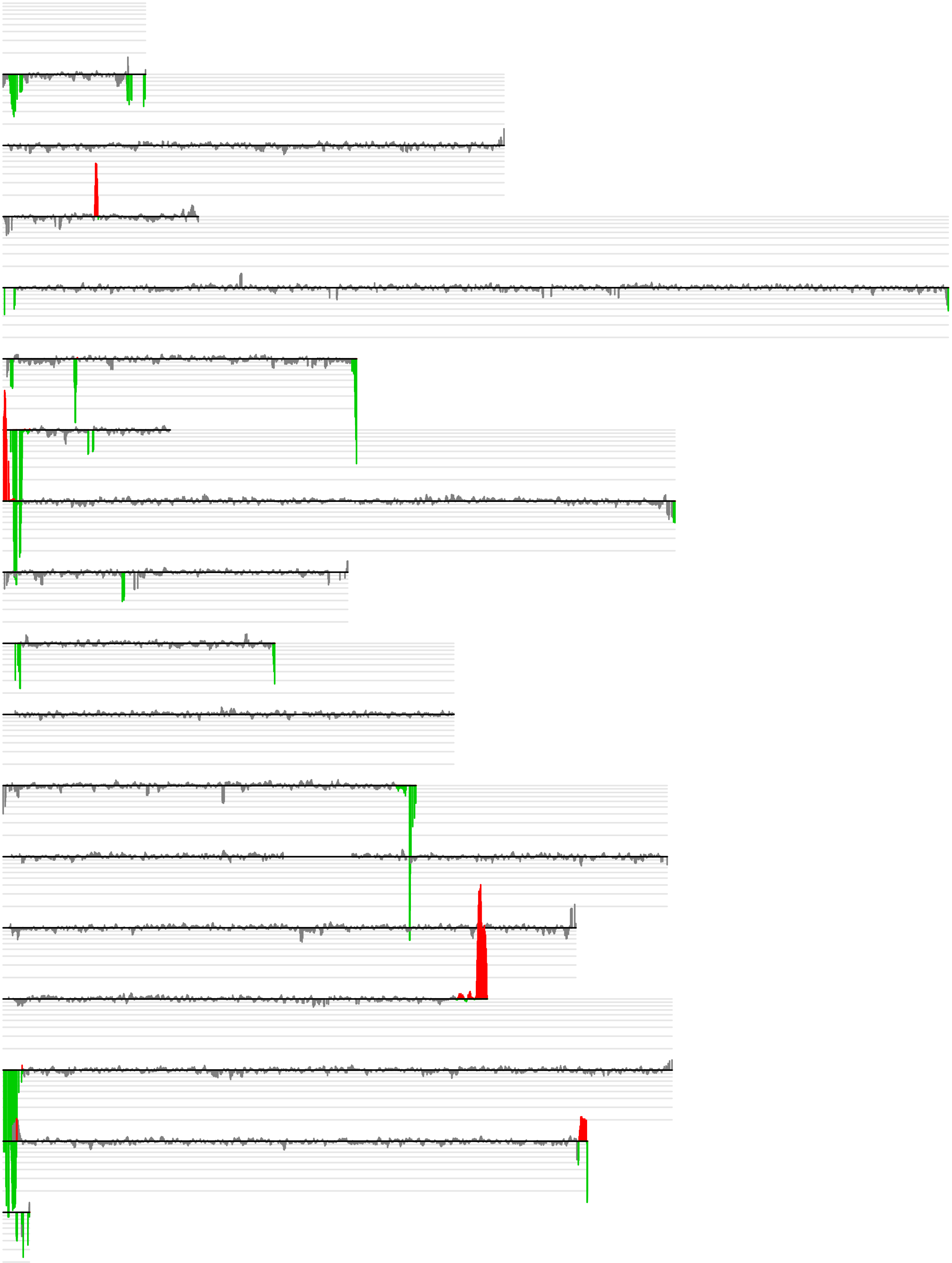

CLAC Plot for Sample: p1 vs PSL2

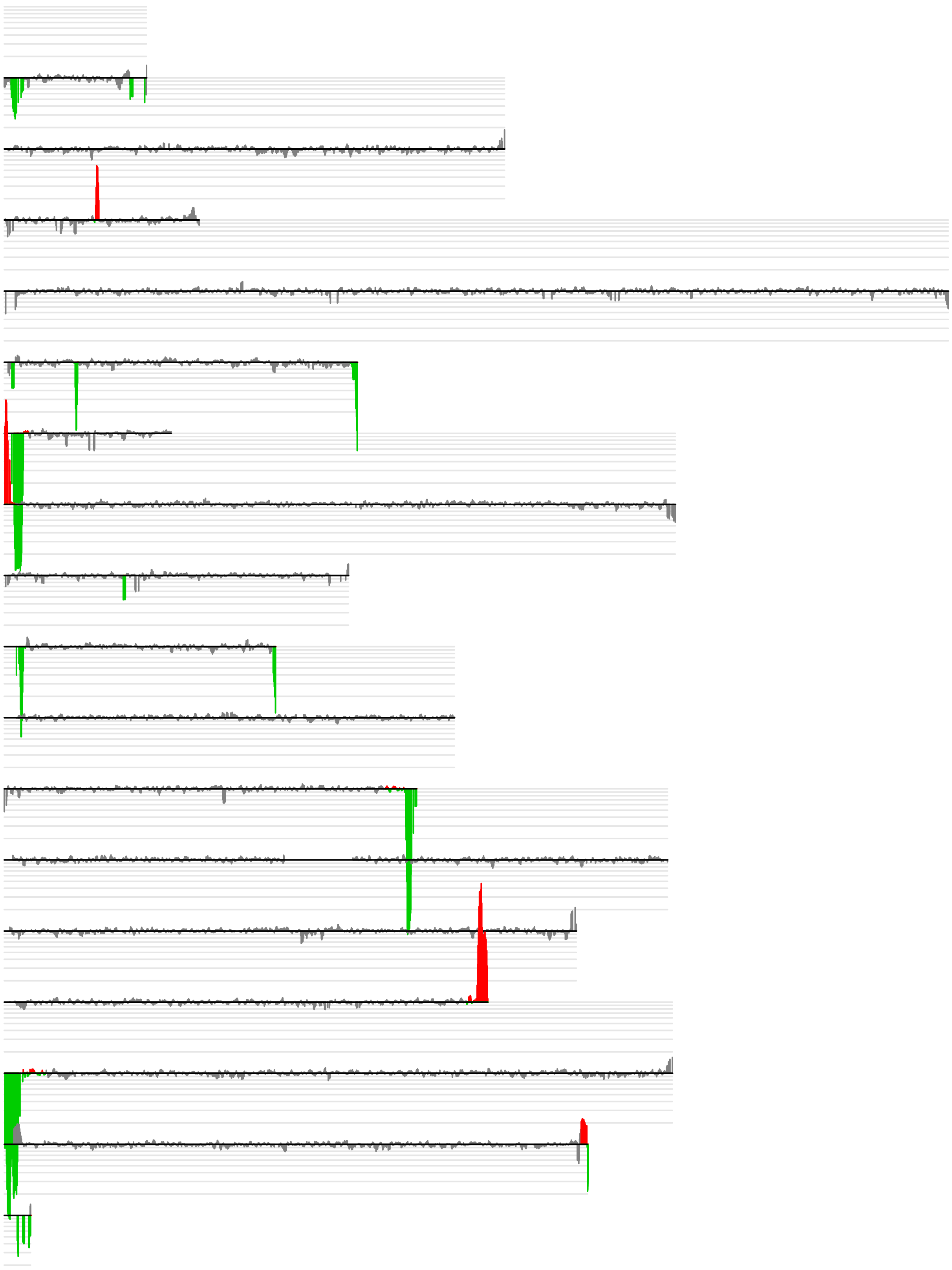

CLAC Plot for Sample: m1 vs PSL2

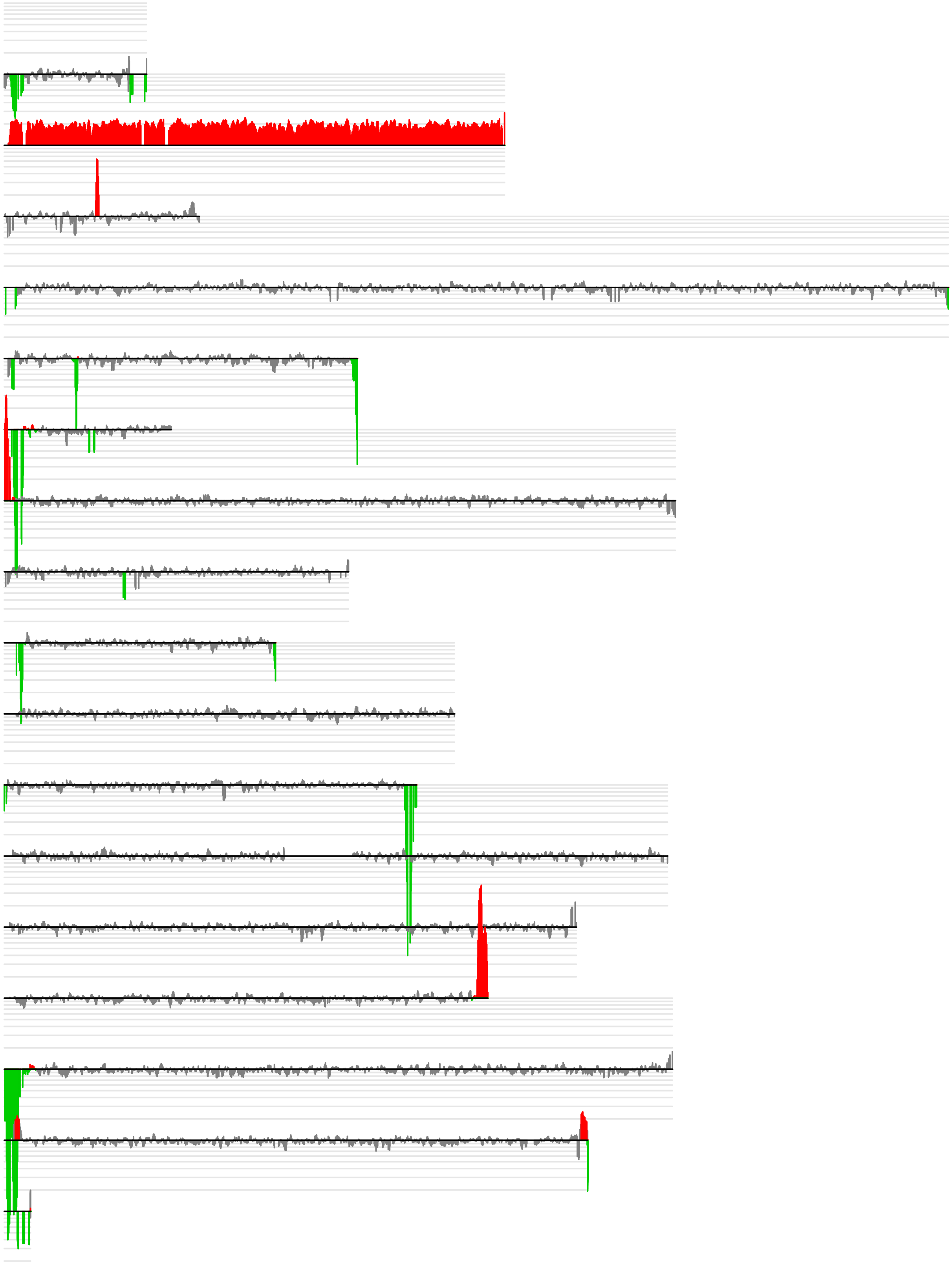

CLAC Plot for Sample: m2 vs PLS2

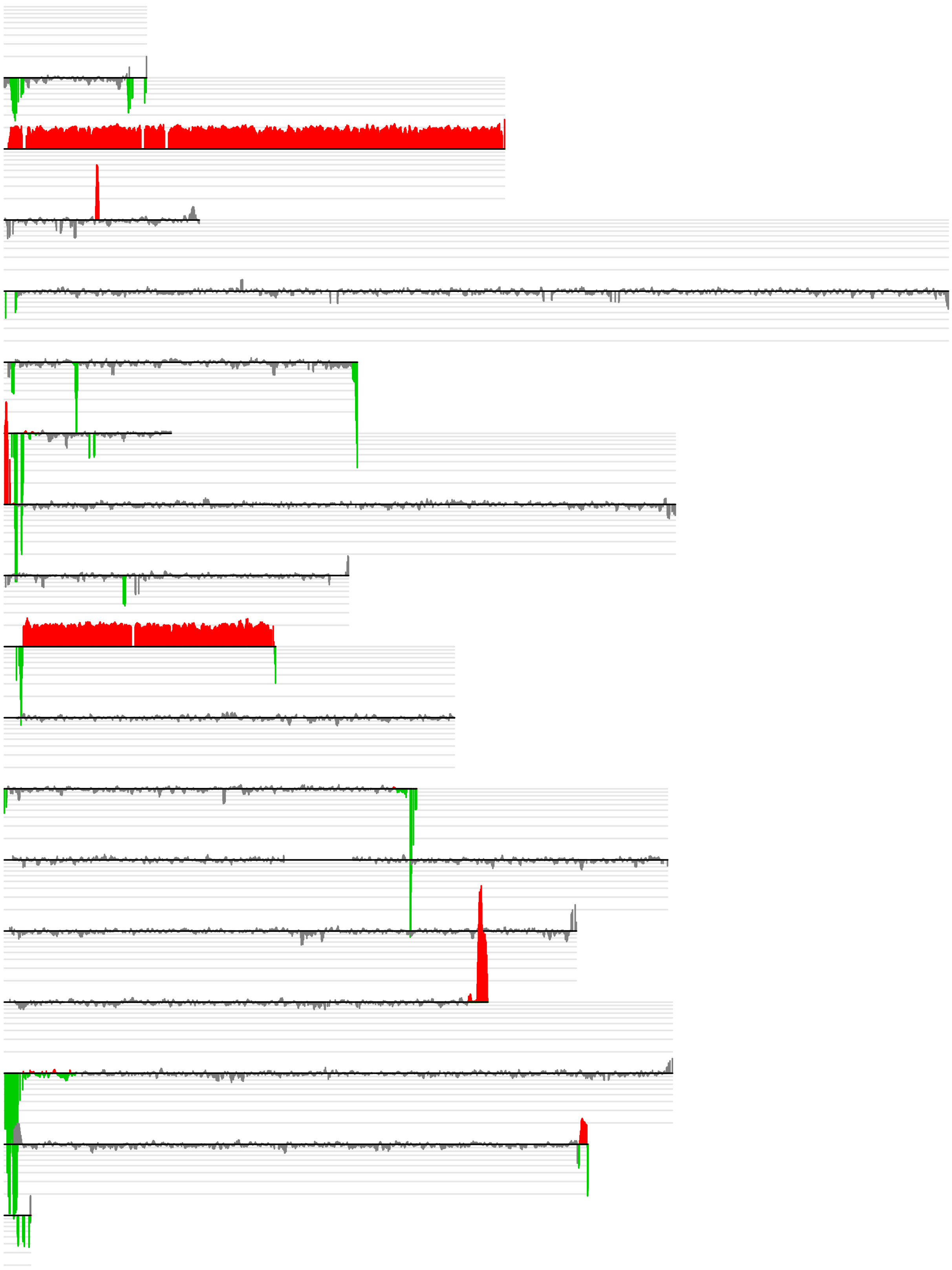

CLAC Plot for Sample: p3 vs PSL2

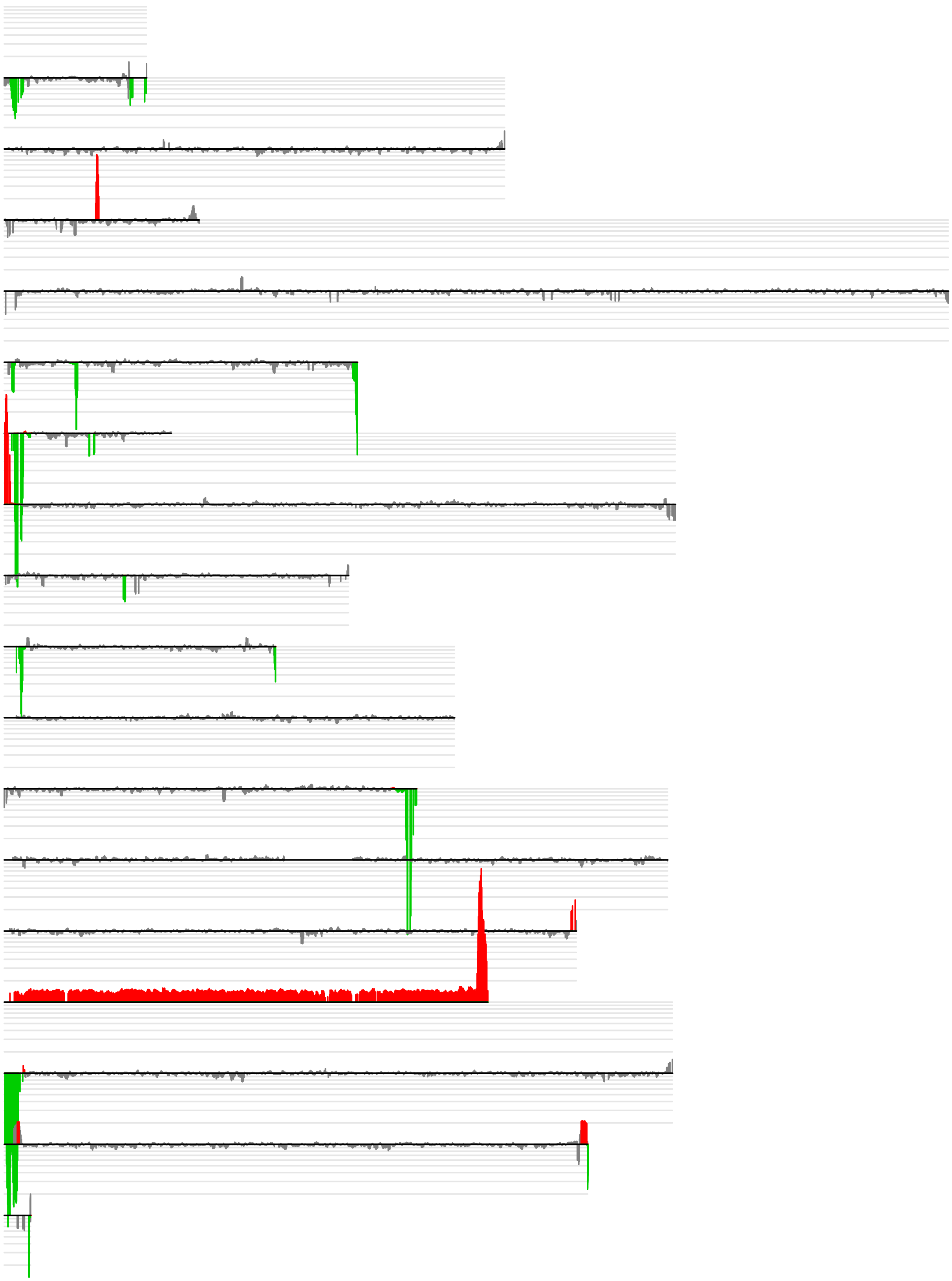

CLAC Plot for Sample: p4 vs p1

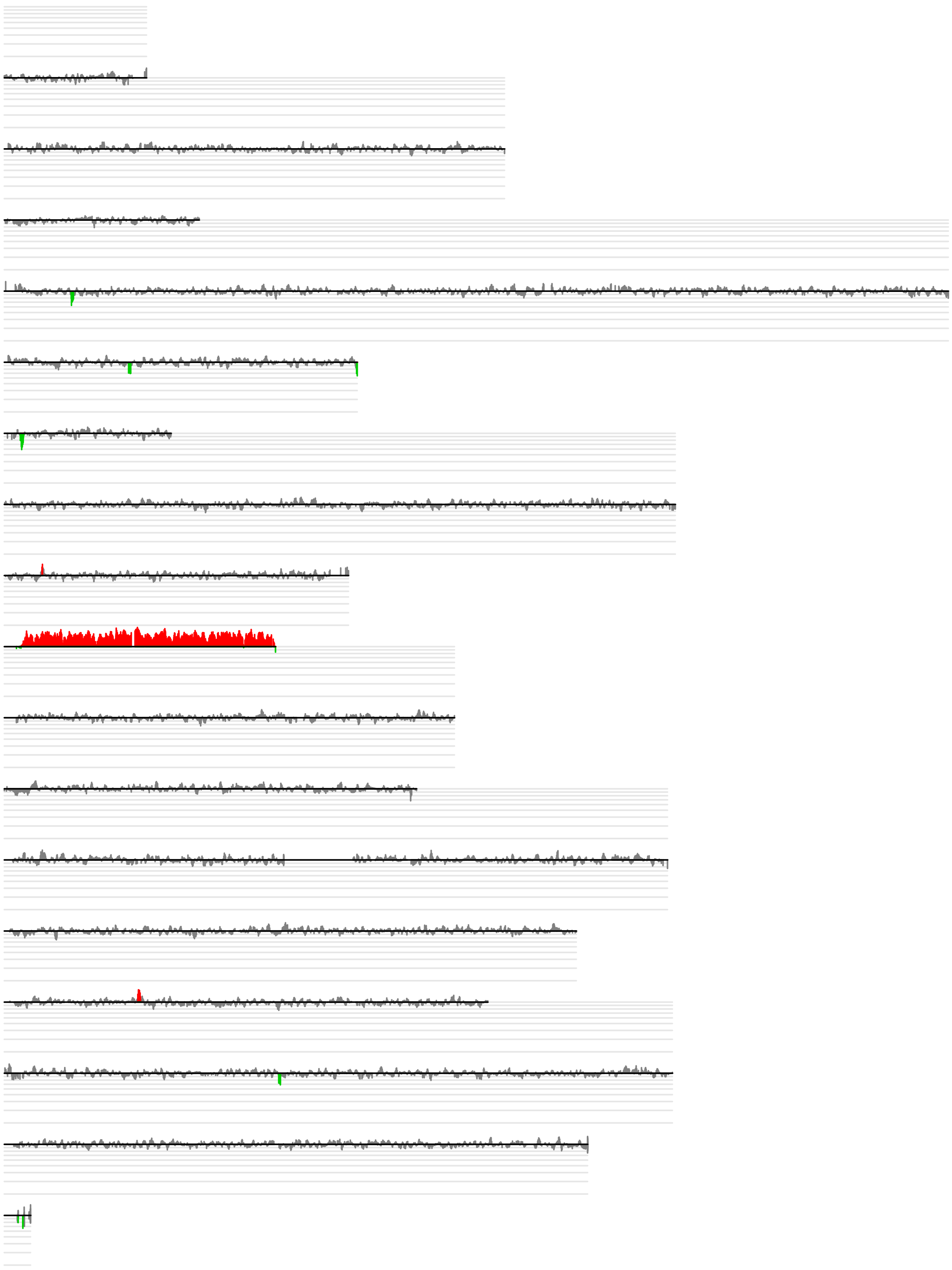

CLAC Plot for Sample: p10 vs p1

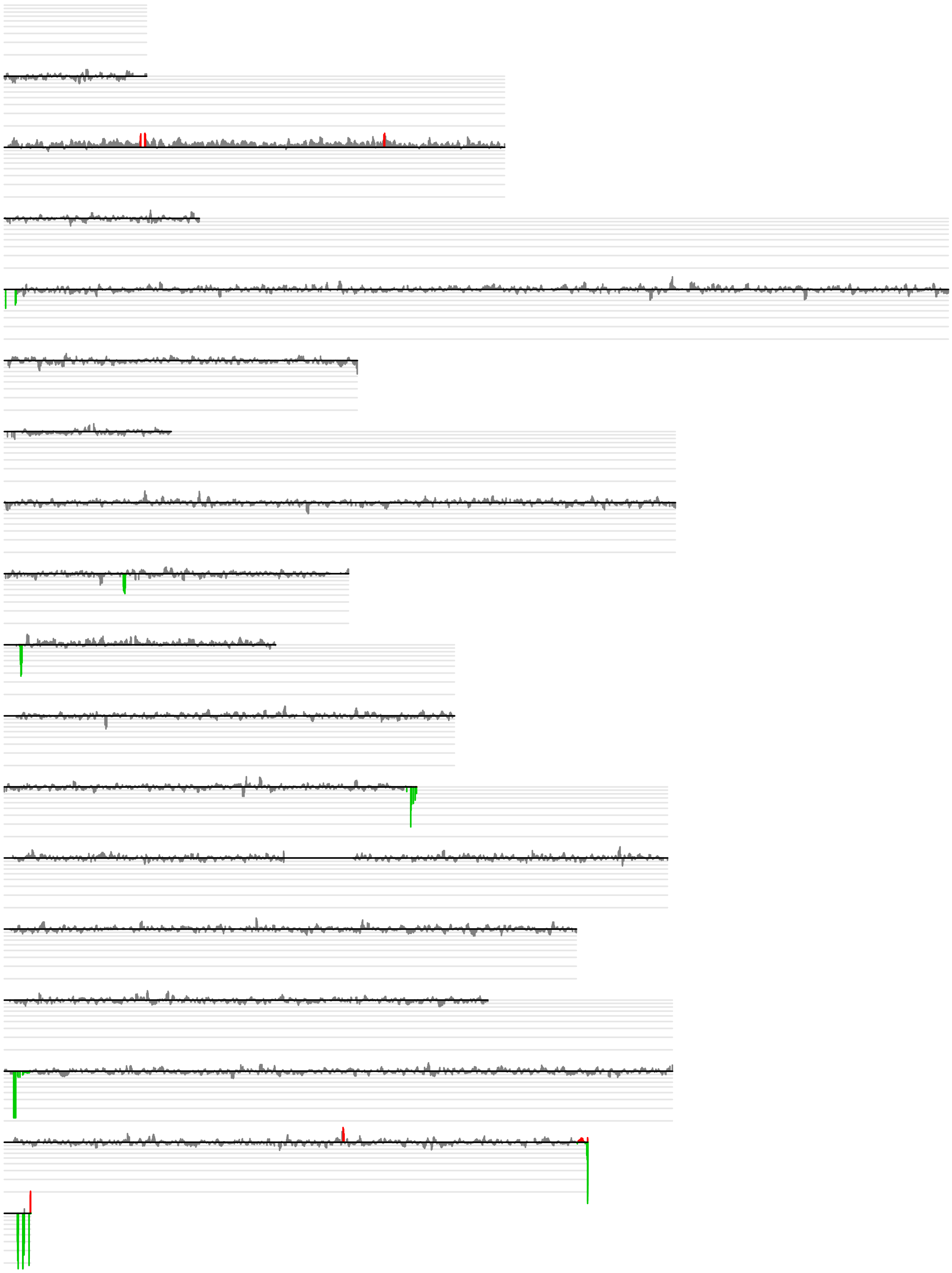

CLAC Plot for Sample: p11 vs p1

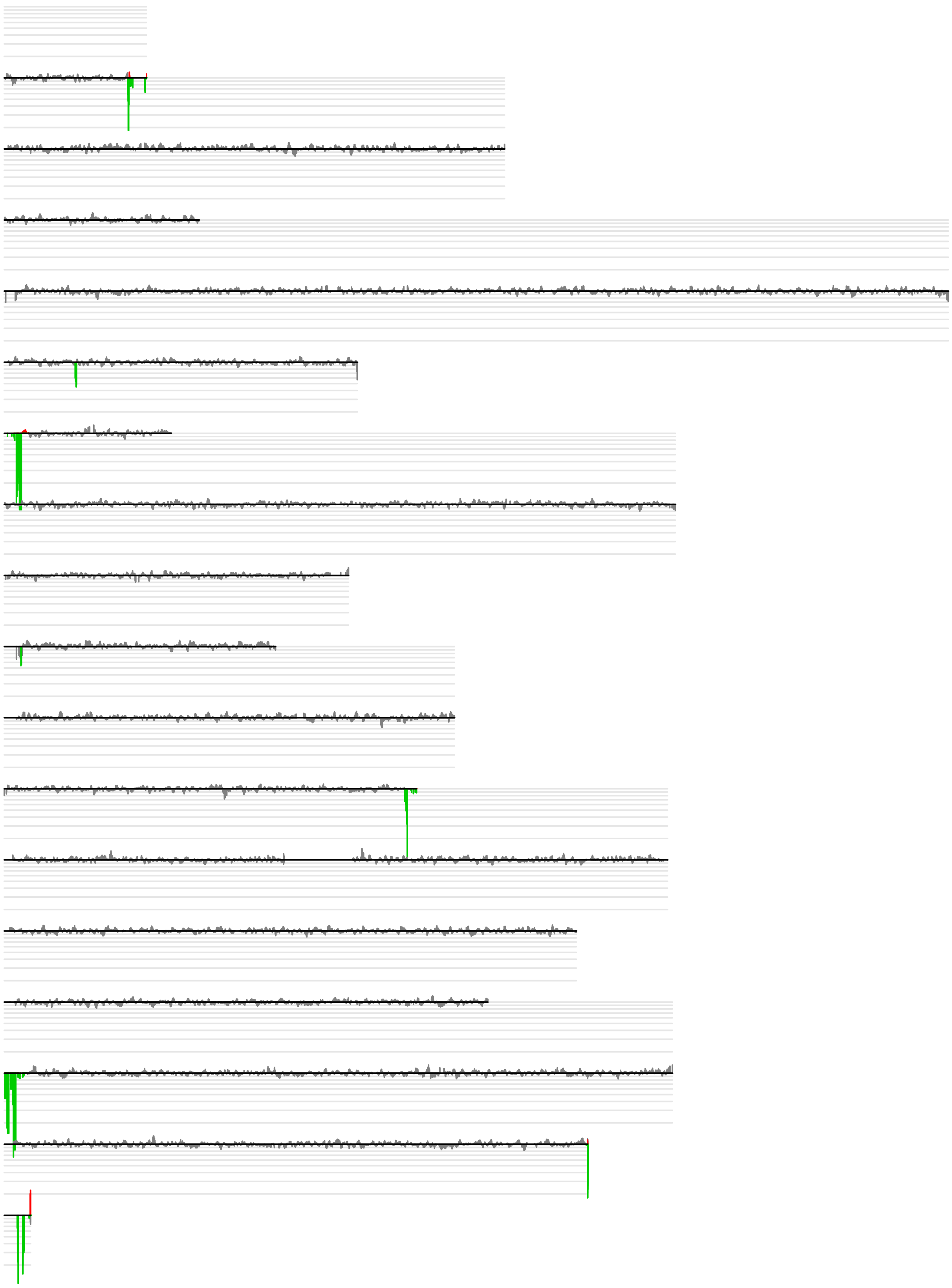

CLAC Plot for Sample: p12 vs p1

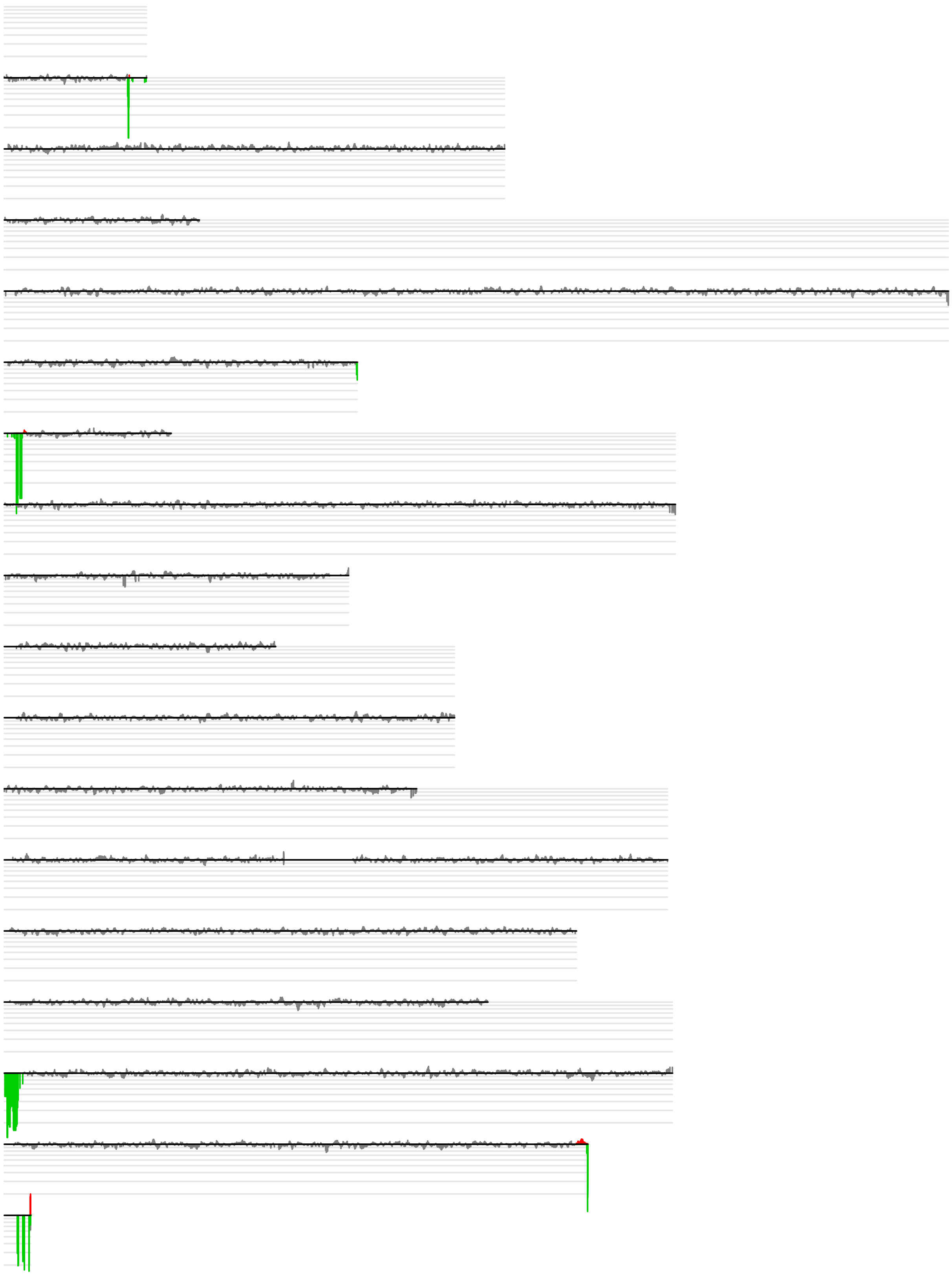

CLAC Plot for Sample: p13 vs p1

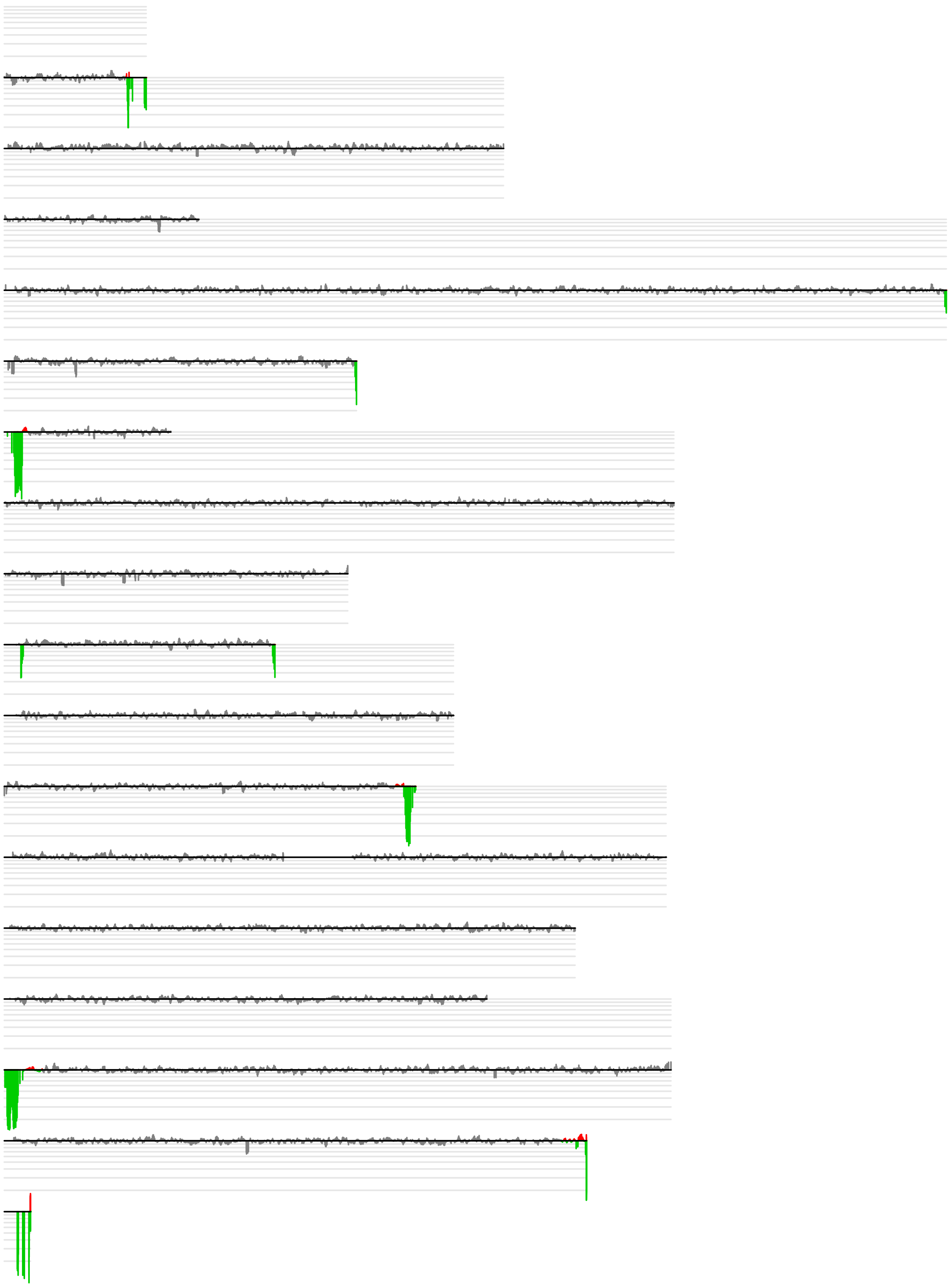

CLAC Plot for Sample: m4 vs p1

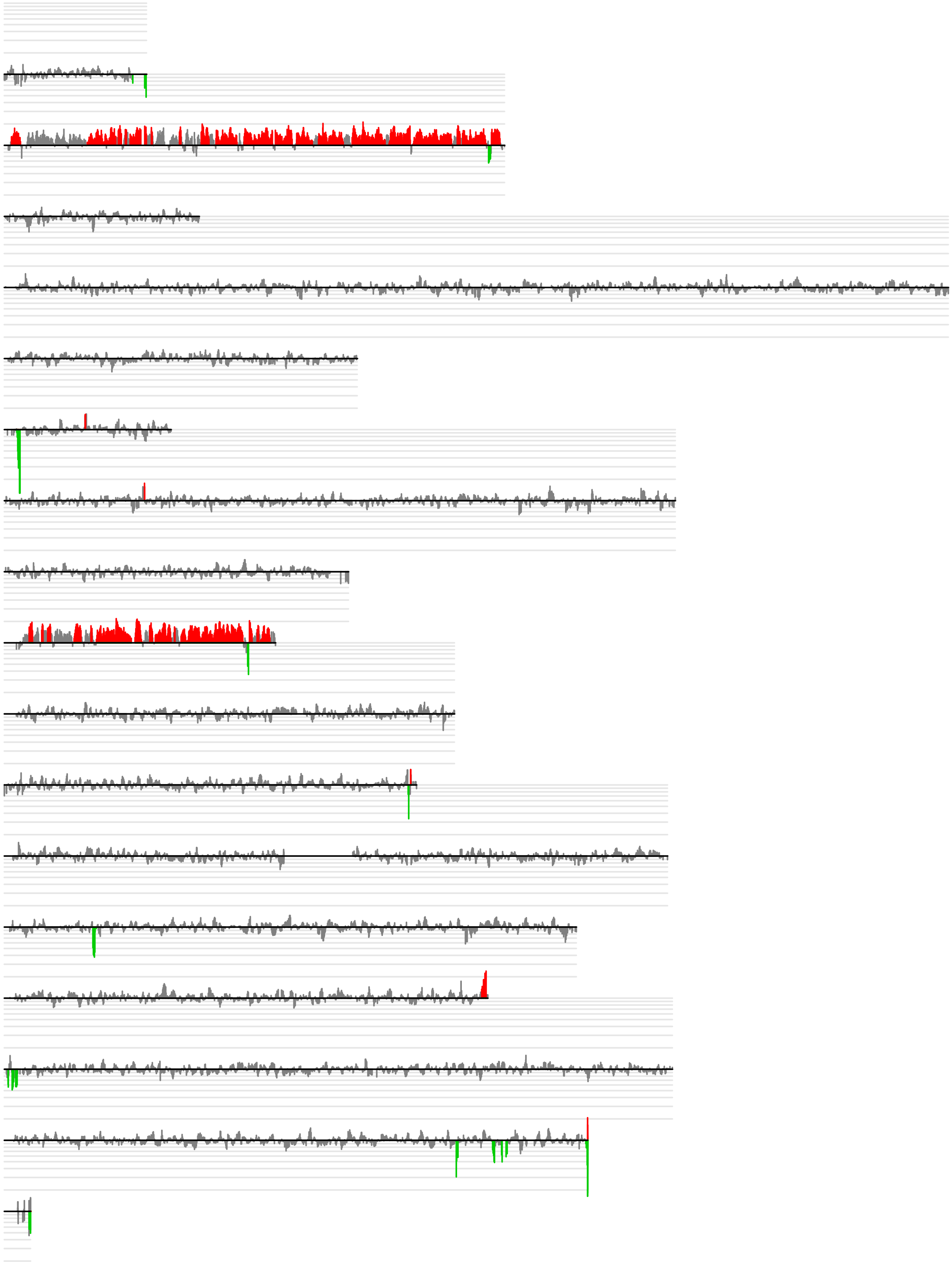

CLAC Plot for Sample: m6 vs p1

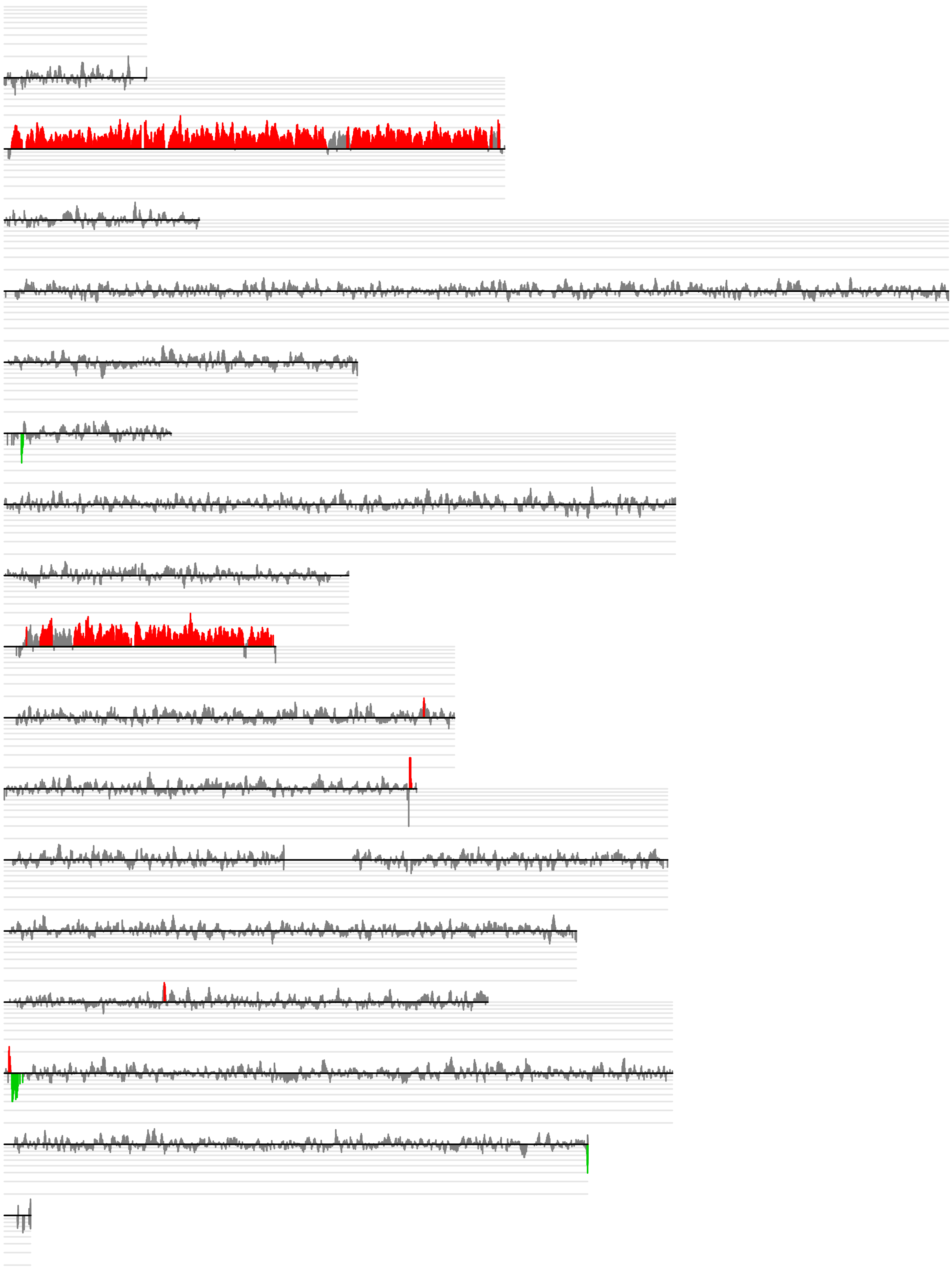

CLAC Plot for Sample: m7 vs p1

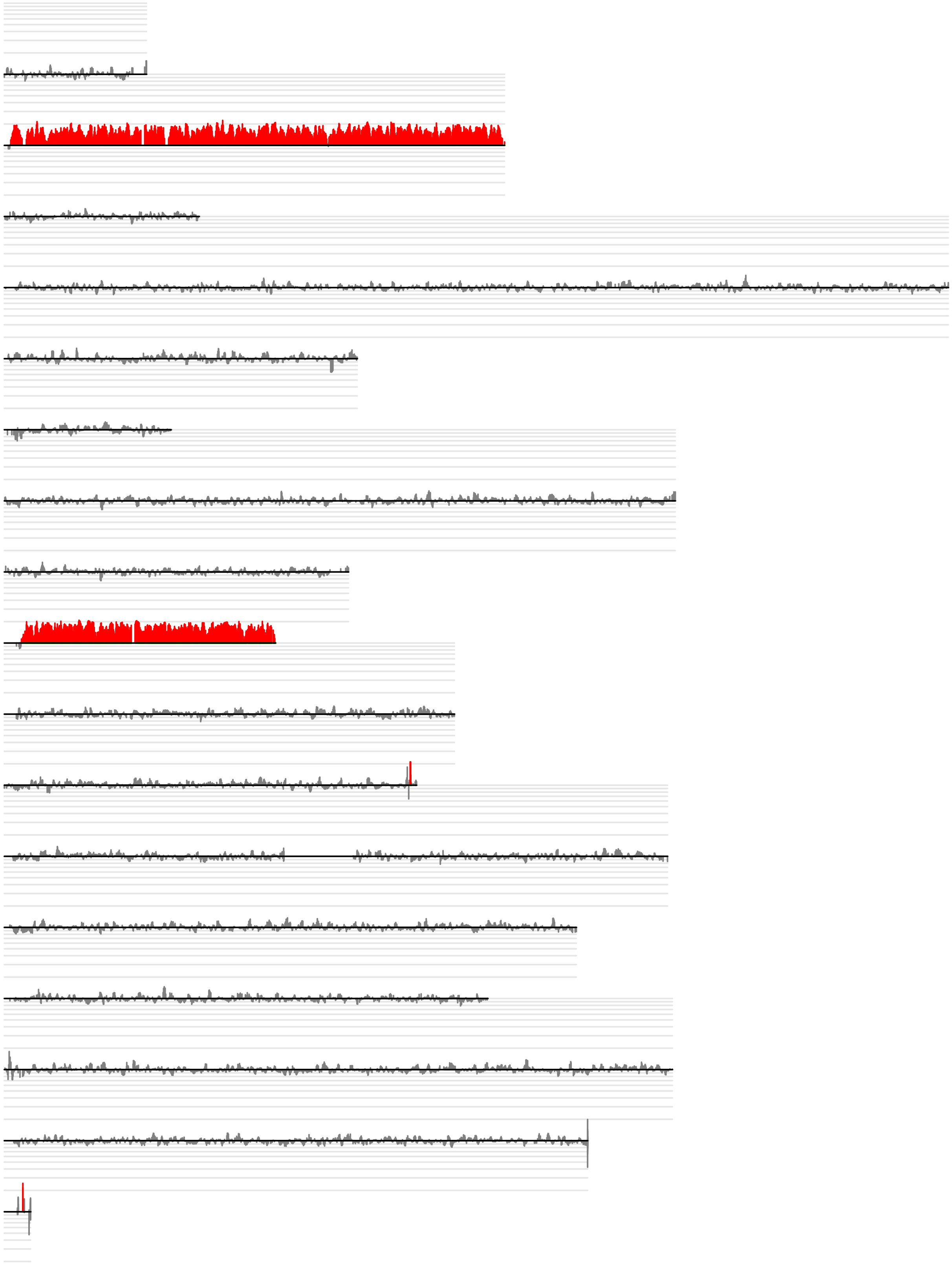

CLAC Plot for Sample: m8 vs p1

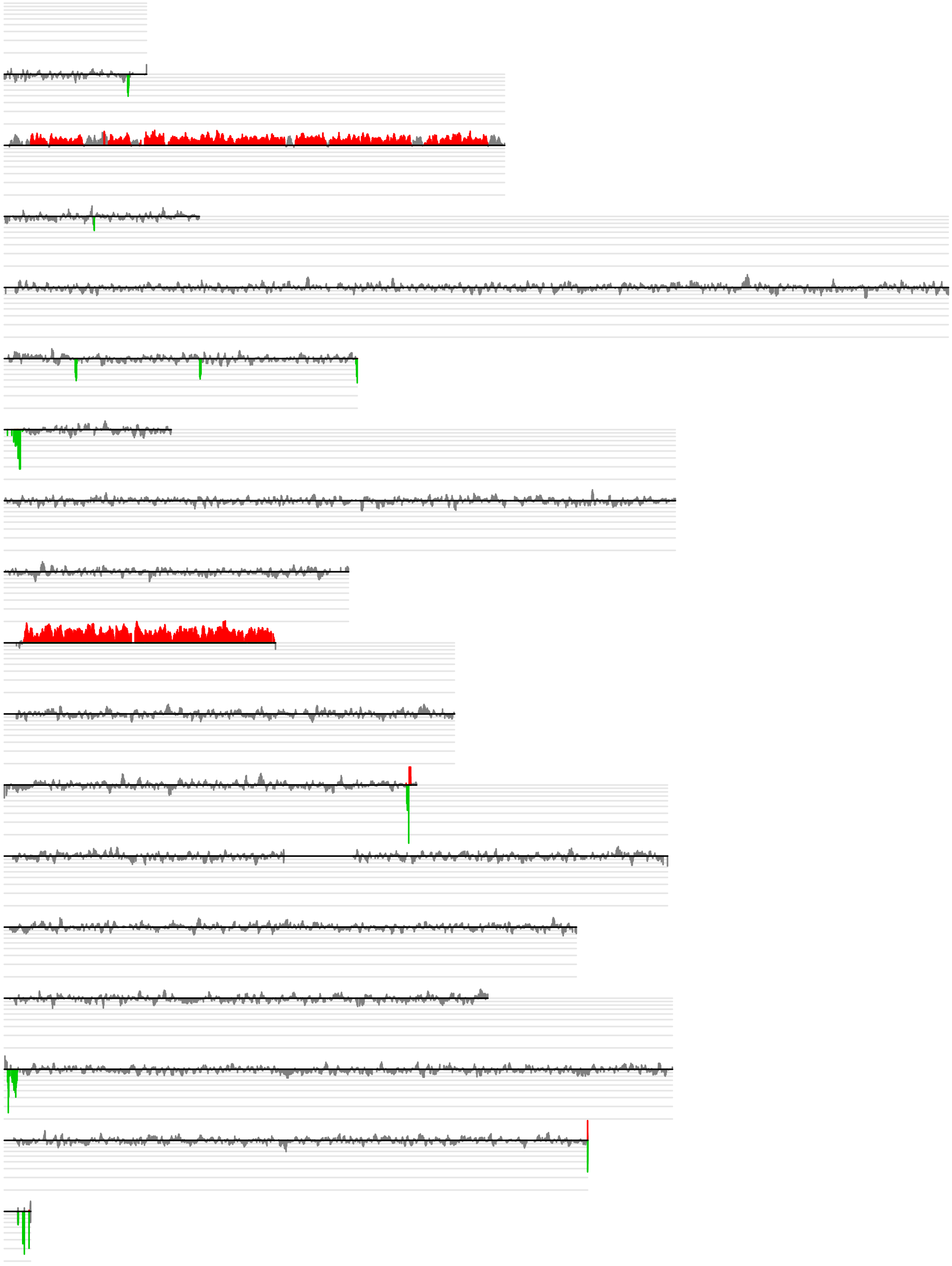

CLAC Plot for Sample: m9 vs p1

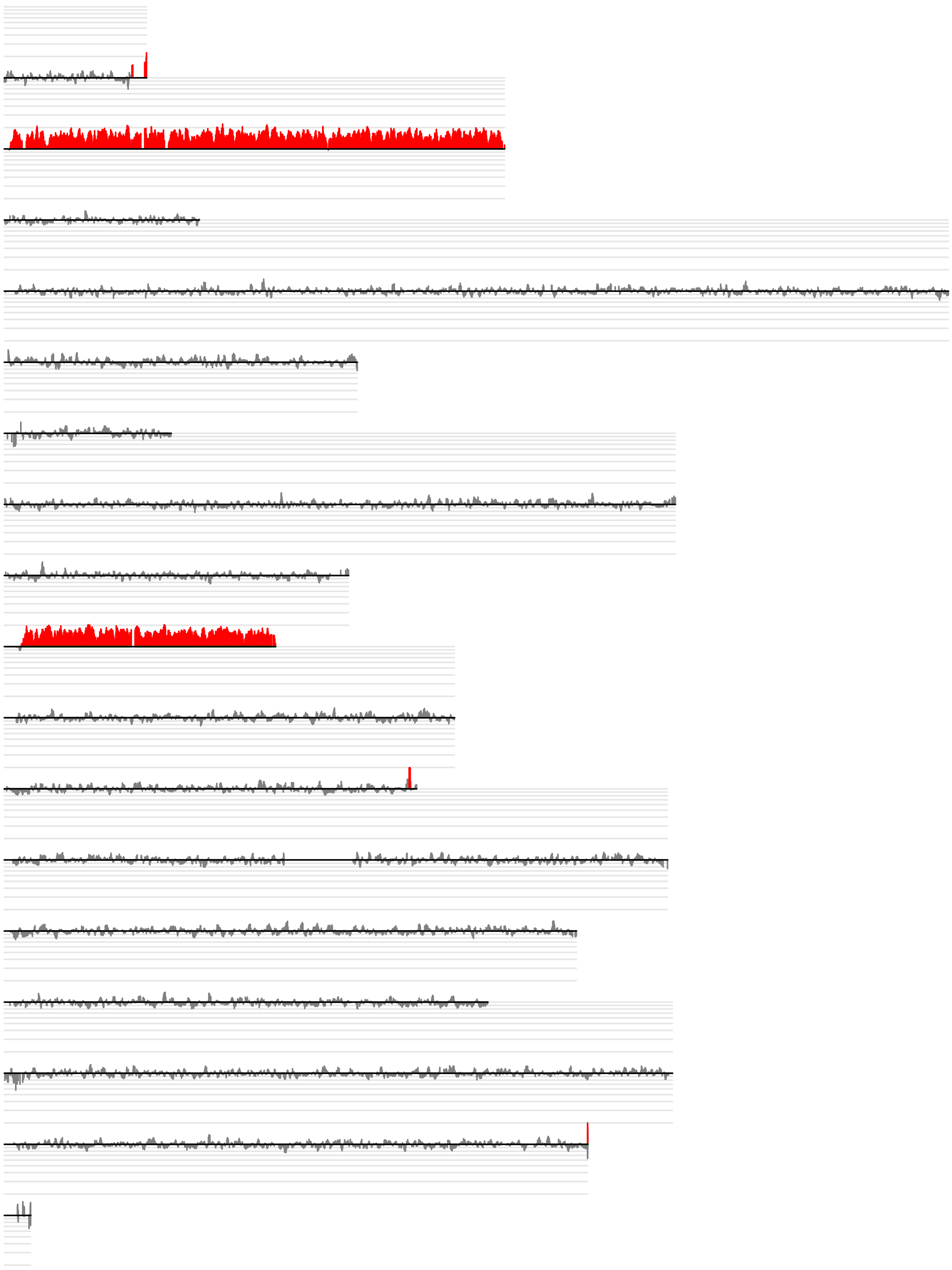

CLAC Plot for Sample: m11 vs p1

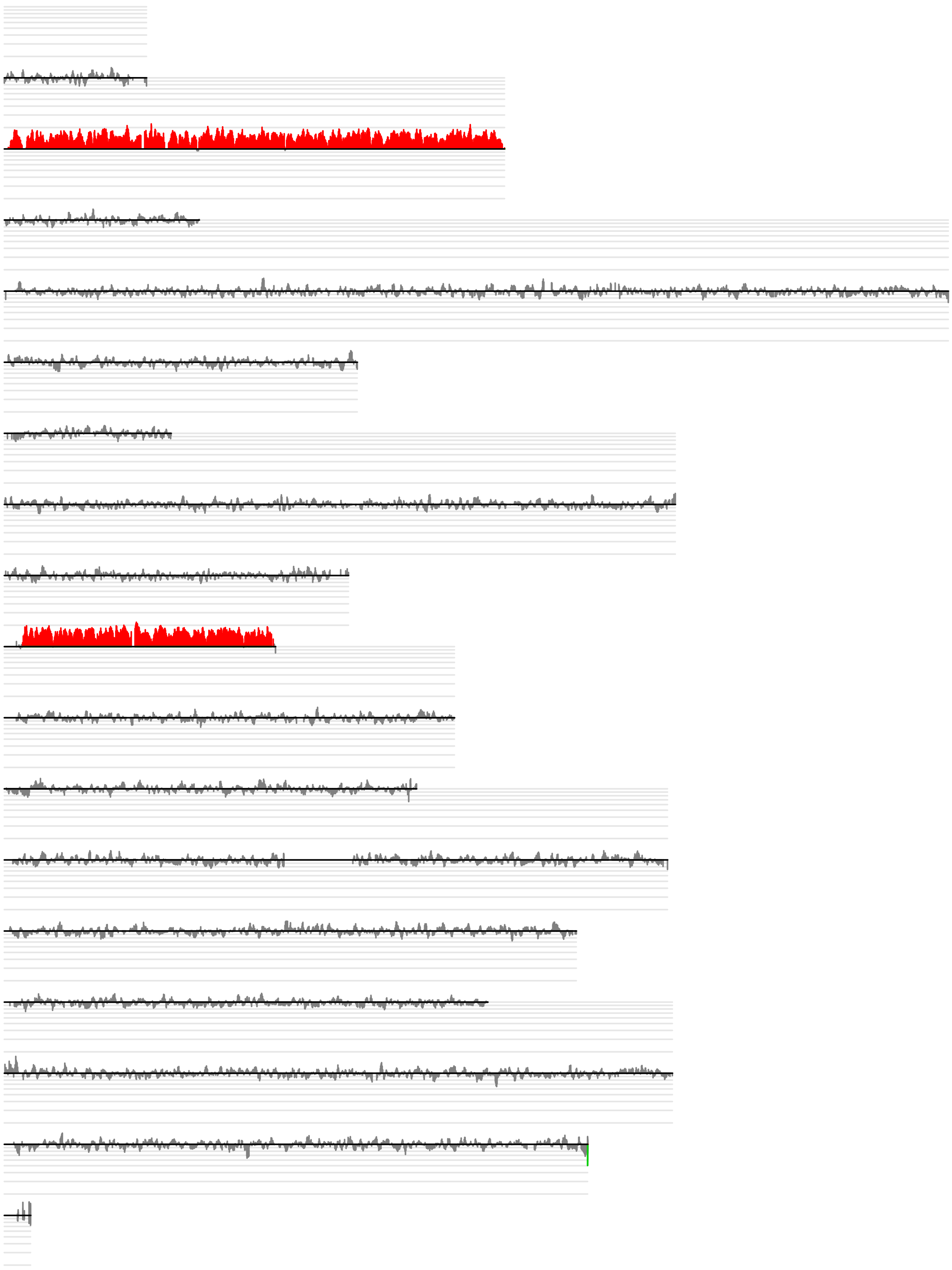

Supplement: Additional file 3: Figure S2. — Graphical summary of aCGH results for each isolate tested. (a) Schematic represenation of the results combined with the information on relationship of the strains (see Fig. 2). aCGH, microarray-based comparative genomic hybridization. WGS, whole genome sequencing. (b) Summarized data for each isolate. Horizontal lines represent chromosomes; red signifies amplified regions while green signifies deleted regions. Clones or strains used as experimental and control samples are indicated on the top of each page. (PDF 3621 kb) [file 12863_2016_464_MOESM3_ESM.pdf]
